# Supplementary material for: Synthetic energy sensor AMPfret deciphers adenylate-dependent AMPK activation mechanism
Source: Nat Commun. 2019 Mar 4;10:1038. doi: 10.1038/s41467-019-08938-z (PMC6399333; doi:10.1038/s41467-019-08938-z)
Supplement: Supplementary file 2 — Supplementary Information [file 41467_2019_8938_MOESM2_ESM.pdf]

Synthetic Energy Sensor AMPfret Deciphers Adenylate-dependent AMPK Activation Mechanism

Martin Pelosse[a,b,c], Cécile Cottet-Rousselle[a], Cécile Bidan[d], Aurélie Dupont[d], Kapil Gupta[c], Imre Berger[c] and Uwe Schlattner[a]

[a] Univ. Grenoble Alpes and INSERM U1055, Laboratory of Fundamental and Applied Bioenergetics (LBFA) and SFR Environmental and Systems Biology (BEeSy), Rue de la Piscine, Domaine Universitaire Saint Martin d'Hères, 38610 Gières, France

[b] European Molecular Biology Laboratory, 71 Avenue des Martyrs, 3800 Grenoble CEDEX, France

[c] Bristol Synthetic Biology Centre BrisSynBio, Biomedical Sciences, University of Bristol, 1 Tankard's Close, Clifton BS8 1TD, United Kingdom

[d] Univ. Grenoble Alpes, CNRS, Laboratoire Interdisciplinaire de Physique (LIPhy), 140 Rue de la Physique, 38402 Saint-Martin-d'Hères, France

- Supplementary data -

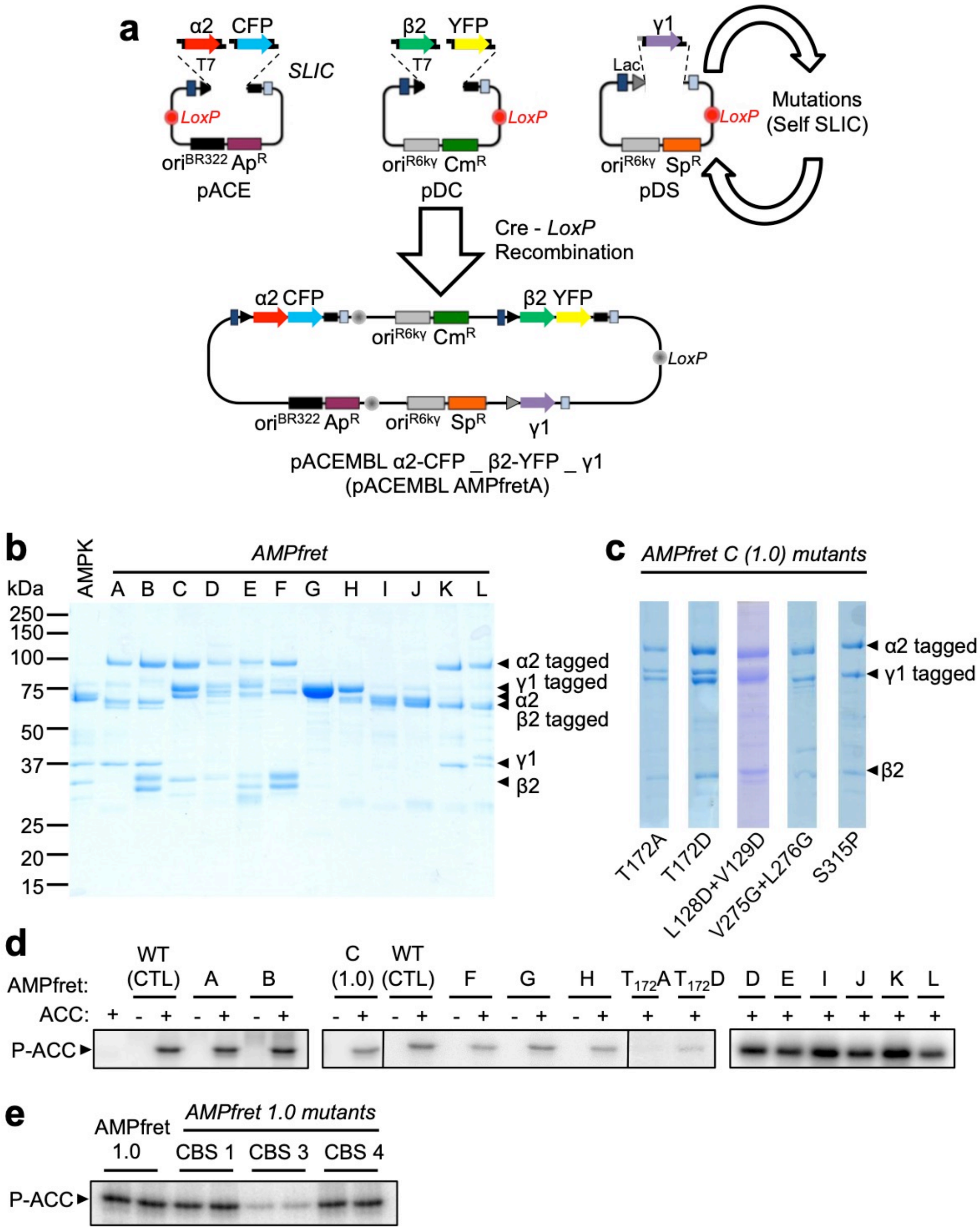

Supplementary Figure 1: Cloning, purification and kinase activity of AMPfret constructs. **(a)** Cloning strategy of AMPfret in the ACEMBL MultiColi expression system. AMPK subunit genes (α2, β2 and γ1 genes depicted by red, green and purple arrows, respectively) flanked or not by a fluorescent protein (CFP and YFP genes depicted by blue and yellow arrows, respectively) were cloned into the ACEMBL acceptor and donor vectors using SLIC or conventional cloning methods. Acceptor and donors were assembled by Cre recombination to generate a single expression plasmid coding for AMPfret heterotrimeric protein complex. **(b)** Purified 12 initial AMPfret constructs separated by SDS-PAGE, AMPK indicates untagged α2β2γ1 complex as control. AMPK subunits are indicated: α2 = 65 kDa, β2 = 30 kDa, γ1 = 37 kDa, eCFP = 27 kDa and YFP = 27 kDa. **(c)** Purified AMPfret C (1.0) mutants separated by SDS-PAGE. Mutations are mentioned below lanes. The three most intense bands correspond to AMPK subunits tagged or not with fluorescent proteins; lanes from different gels. **(d)** Kinase activity of AMPfret constructs. Autoradiogram of acetyl-CoA carboxylase (ACC) phosphorylated by native AMPK (WT) and initial AMPfret constructs in presence of 200 µM ^{32}P-labelled ATP and 40 µM AMP. AMPfret constructs (A-L) and AMPfret C (1.0) derived mutants T172A and T172D. Differences in intensity are inherent to assay conditions like exposure time. **(e)** Kinase activity of AMPfret 1.1 and derived CBS site mutants; all conditions like before.

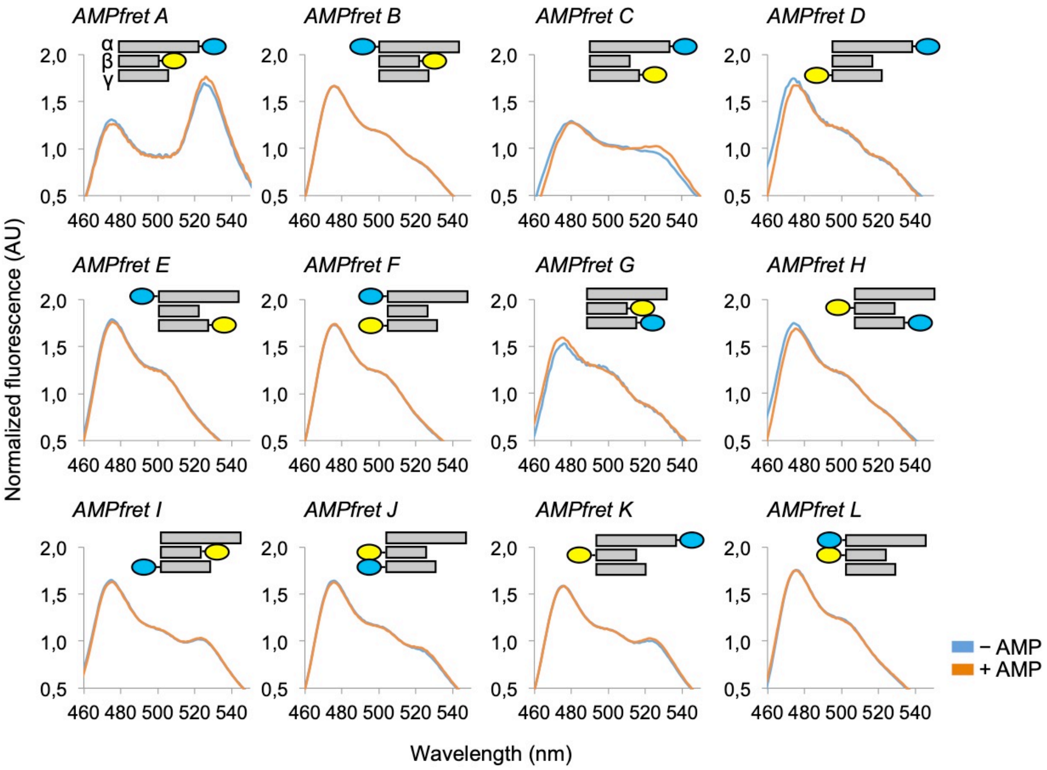

Supplementary Figure 2 Topology and fluorescence emission spectra of AMPfret A - L. The small sketch above each spectrum represents the topology of the AMPfret fusion construct (CFP and YFP are represented as blue and yellow circles, respectively). Purified proteins were incubated in presence of 3 mM freshly prepared ATP (blue line), or in presence of 20 µM AMP (orange line). Peaks at 476 and 527 nm correspond to the emission peaks of CFP and YFP, respectively. Spectra in presence of ATP or AMP were normalized using the CFP/YFP pair isosbestic point at 512 nm.

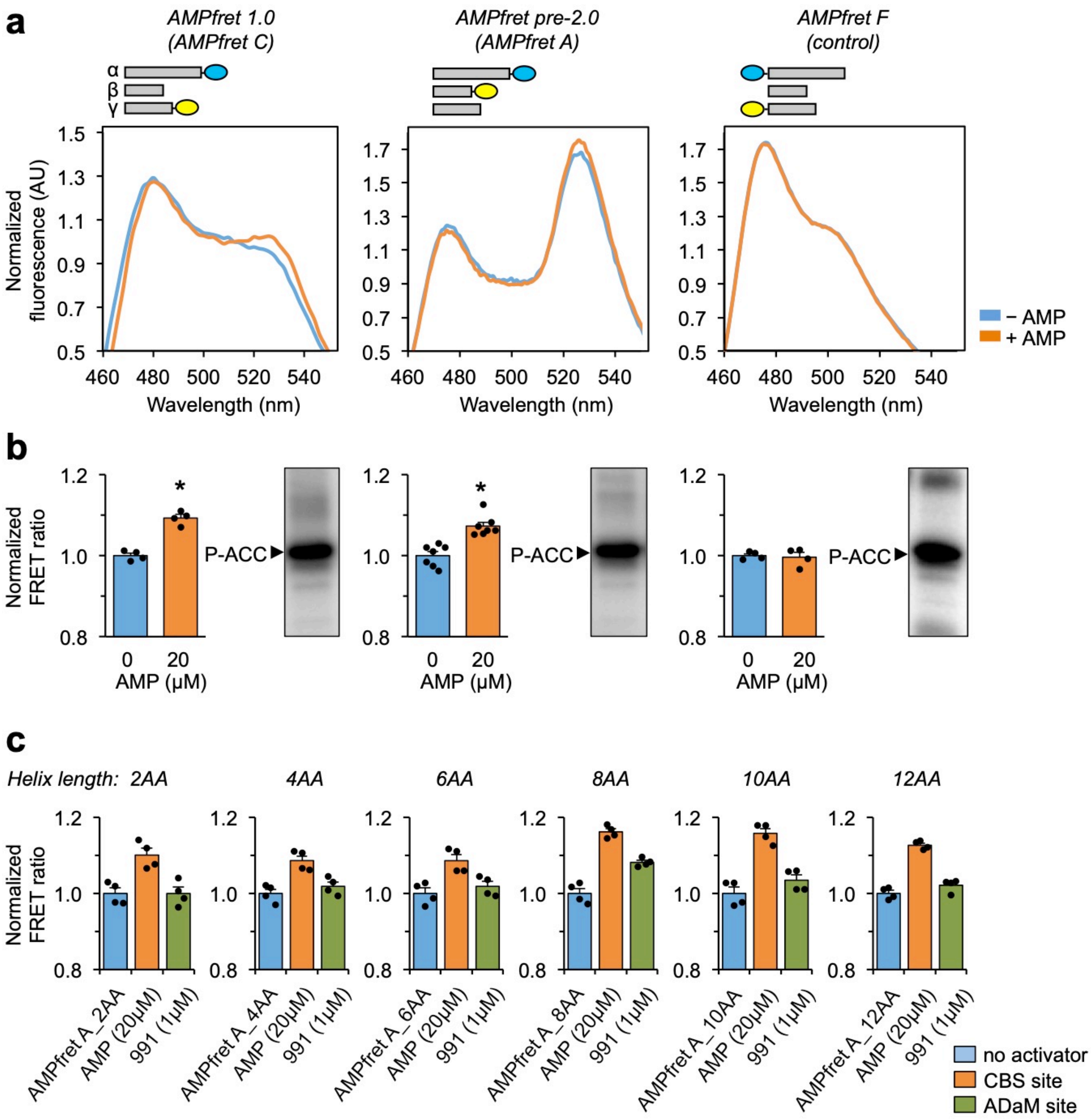

Supplementary Figure 3 Topology, fluorescence emission spectra and activity of AMPfret C (1.0) and AMPfret A (pre-2.0), the initial two positive constructs that show significant FRET ratio difference upon AMP binding, and a representative negative construct, AMPfret F. **(a)** Topology of the AMPfret construct (CFP and YFP are represented as blue and yellow circles, respectively) and corresponding fluorescence spectra. Purified proteins were incubated in presence of 3 mM freshly prepared ATP (blue), or in presence of 20 µM AMP (orange) in buffer containing 5 mM $MgCl_2$. Spectra in presence of ATP or AMP were normalized using the CFP/YFP isosbestic point at 512 nm. **(b)** FRET ratio of AMPfret constructs calculated from data in (a) (same color code). FRET ratio (CFP/YFP) was normalized to the incubation in presence of ATP. Autoradiograms of *in vitro* kinase activity assays using acetyl-CoA carboxylase (ACC) as a substrate are also depicted. Data and error bars correspond to mean ± SEM (n ≥ 4; *p < 0,001, Student's t-test). **(c)** Optimization of AMPfret. Helices of different length (2 to 12 amino acids) were inserted as a linker between α2 and CFP. Once purified, these constructs were screened for improved AMP-induced (orange) and 991-induced FRET change (green). Data are normalized according to the FRET ratio in absence of any compound (blue). Data and error bars correspond to mean ± SEM (n = 4).

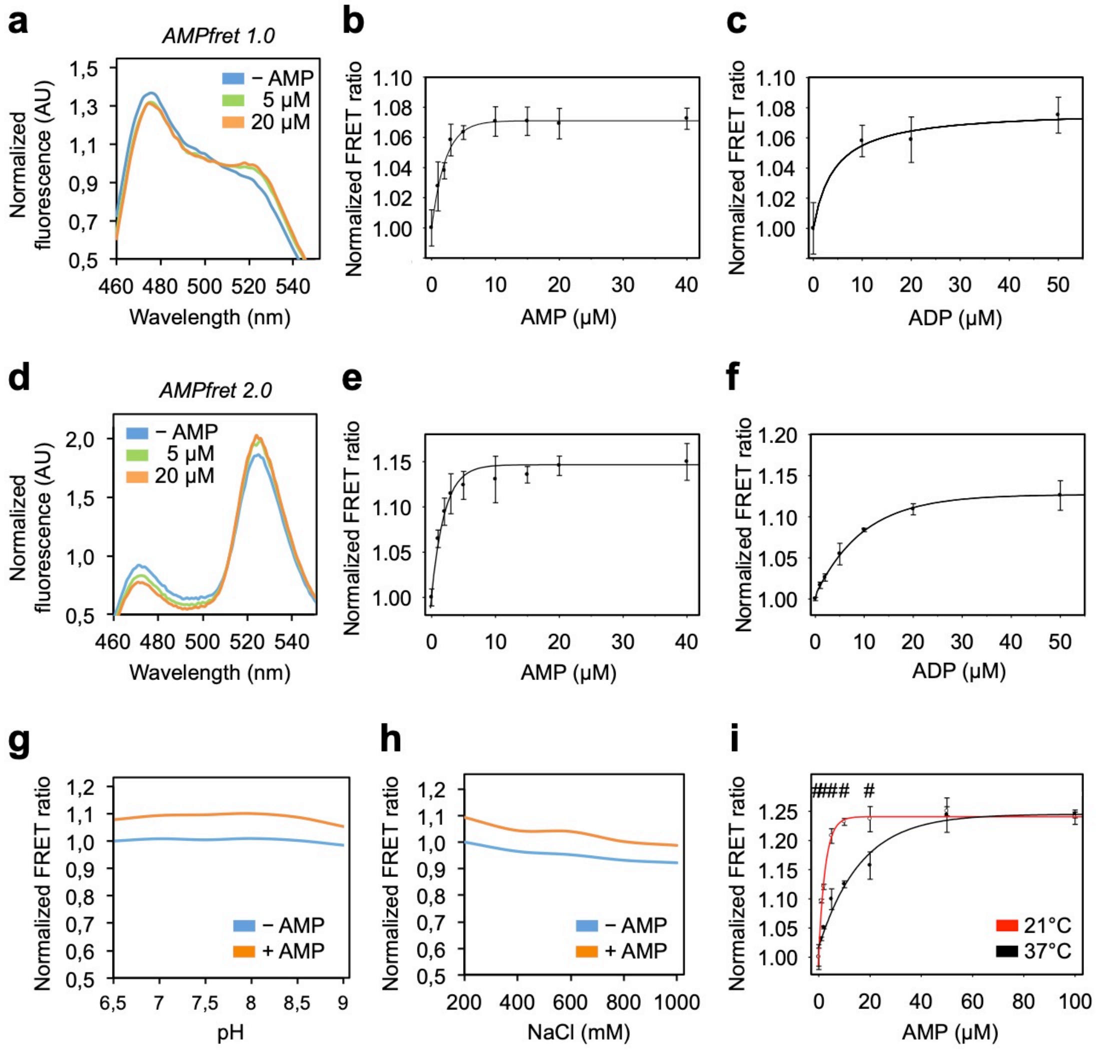

Supplementary Figure 4 AMPfret responses to AMP, ADP, pH, salt and temperature. **(a-c)** AMPfret 1.0 signal upon increasing AMP or ADP concentrations. **(a)** Fluorescence emission spectrum and its variation upon AMP binding (blue, green and orange lines: no AMP, 5 µM and 20 µM AMP, respectively). **(b)** AMP- dependent FRET ratio calculated from fluorescence emission spectra. Data were fitted to single site binding kinetics, yielding an affinity of 1.8 µM. Data and error bars correspond to mean ± SEM (n=5). **(c)** ADP-dependent FRET ratio calculated from fluorescence emission spectra. Data were fitted to single site binding kinetics, yielding an affinity of about 5 µM. Data and error bars correspond to mean ± SEM (n≥3). **(d-f)** AMPfret 2.0 signal upon increasing AMP or ADP concentrations. **(d)** Fluorescence emission spectrum and its variation upon AMP binding (blue, green and orange lines: no AMP, 5 µM and 20 µM AMP respectively). **(e)** The AMP-dependent FRET ratio of AMPfret 2.0 was calculated from fluorescence emission spectra. Data were fitted to single site binding kinetics, yielding affinity of 1.5 µM. Data and error bars correspond to mean ± SEM (n≥3). **(f)** The ADP- dependent FRET ratio calculated from fluorescence emission spectra. Data were fitted to single site binding kinetics, yielding an affinity constant of about 7.5 µM. Data and error bars correspond to mean ± SEM (n≥3). **(g)** AMPfret 1.1 was incubated in buffers containing 50 mM Tris pH 8, 2 mM β-mercaptoethanol and various amounts of salt (200 mM – 1 M; blue line). The FRET signal variation due to the addition of 20 µM AMP was measured (orange line). **(h)** AMPfret 1.1 was incubated at different pH in buffer containing 200 mM NaCl, 2 mM β-mercaptoethanol, 50 mM Tris (pH 6,5 – 9; blue line). The FRET signal variation due to the addition of 20 µM AMP was measured (orange line). **(i)** The AMP-dependent FRET ratio of AMPfret 2.1 was calculated from fluorescence emission spectra at RT and at 37^{0}C. Data were fitted with Sigma Plot 13.0 to single site binding kinetics, yielding an affinity of 1.5 µM at RT and 8 µM at 37^{0}C. Data and error bars correspond to mean ± SEM ($n \geq 3$; $^{\#}p < 0.05$, Student's t-test).

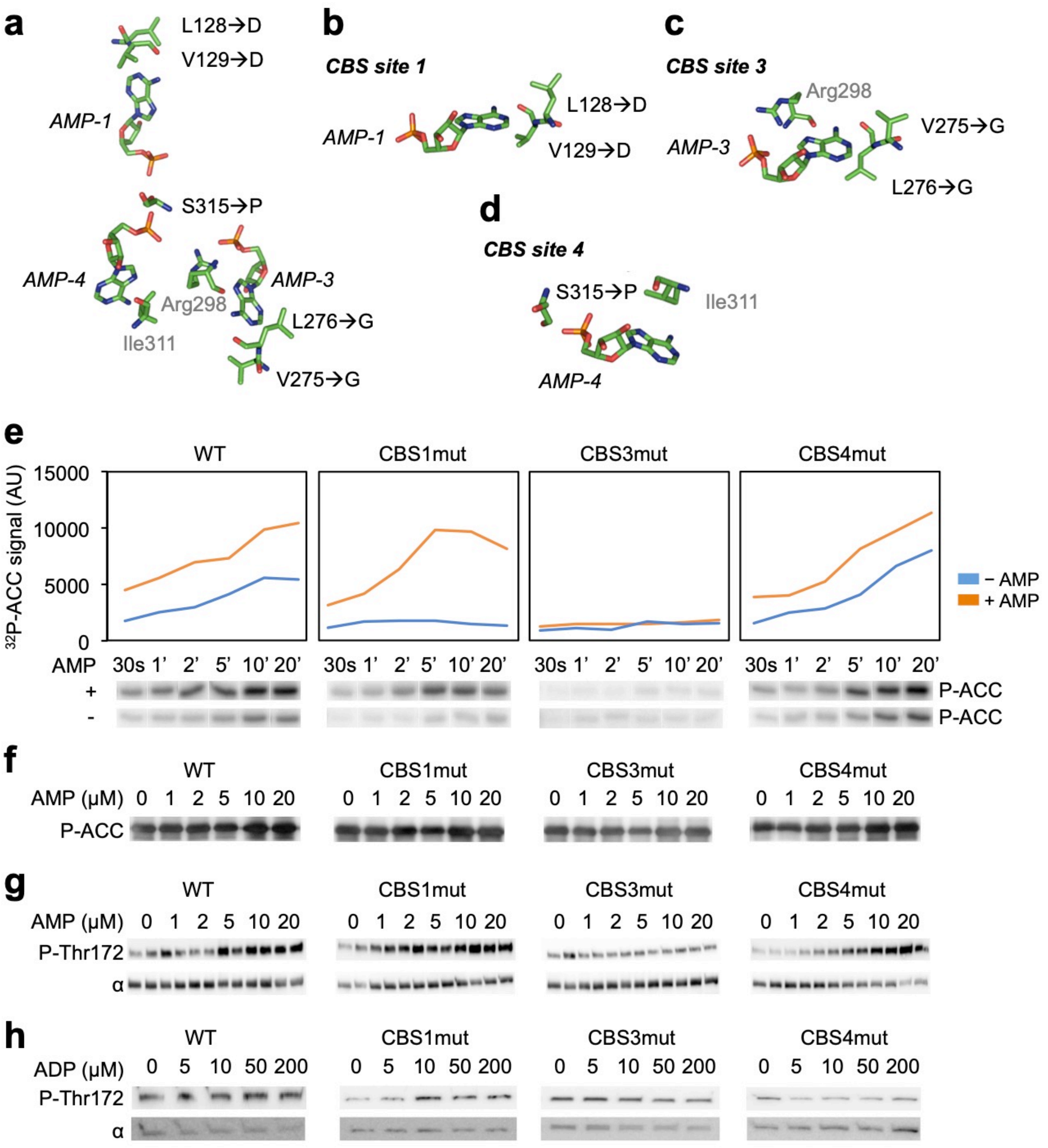

Supplementary Figure 5 (a-d) Residues involved in adenine nucleotide binding at γ1 AMPK CBS sites 1, 3 and 4 (details from PDB ID: 4EAI). AMP moieties are numbered according to the CBS site. Amino acids mutated to selectively inhibit nucleotide binding at the level of specific CBS sites (black) or reported to be involved in nucleotide binding but not mutated (grey). **(a)** Overview of the maximally three AMPs bound to γ1. **(b-d)** Details of the three CBS sites with AMP binding residues. **(e)** Kinetics of allosteric activation by AMP. Kinase assays with pre-activated AMPfret 1.0, wild-type (WT) or CBS site mutants, and acetyl-CoA carboxylase (ACC) as substrate, were done in absence (blue line) or in presence (orange line) of 20 μM AMP and radiolabeled 200 μM ^{32}P-ATP (with 3 pmol of each protein). Autoradiograms of labeled ACC (Typhoon imager, lower panel) and quantification of bands (upper panel) are shown. **(f-h)** AMPfret 1.0 wild-type (WT) and CBS site mutants analyzed for AMPK activation. **(f)** Allosteric activation by AMP. Activity assays in presence of increasing concentrations of AMP (0 – 20 μM) and fixed MgATP (200 μM). AMPK activity was visualized by immunoblotting for P-ACC. **(g)** AMP-dependent protection against dephosphorylation by PP2Cα in presence of increasing concentrations of AMP (0 – 20 μM). Protection against dephosphorylation was visualized by immunoblotting for P-T172 AMPK. **(h)** ADP-dependent protection against dephosphorylation by PP2Cα in presence of increasing concentrations of ADP (0 – 200 μM). Protection against dephosphorylation was visualized by immunoblotting for P-T172 AMPK.

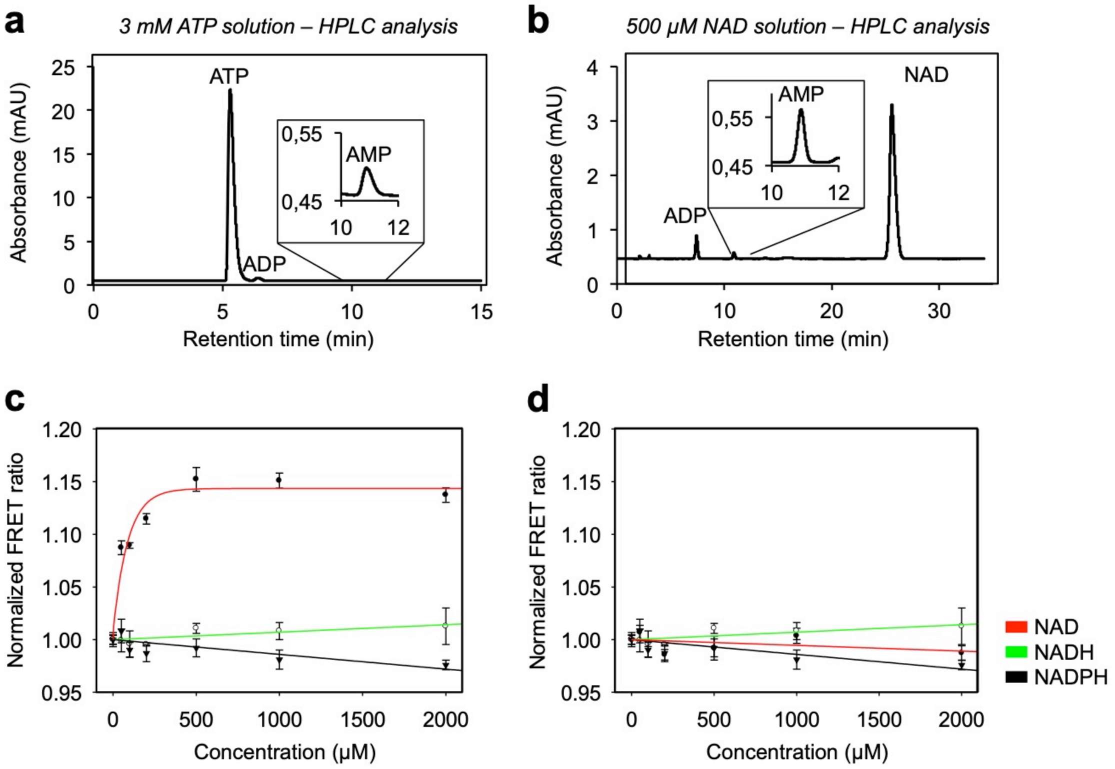

Supplementary Figure 6 (a) HPLC elution profile (C-18 column) of freshly made 3 mM ATP solution. Note the presence of AMP (retention time = 10,9 min). According to a calibration curve (not shown), this peak corresponds to 5 µM AMP. **(b)** HPLC elution profile (C-18 column) of freshly made 500 µM NAD solution, showing again the presence of AMP. According to a calibration curve (not shown), this peak corresponds to 12,5 µM AMP. **(c)** AMPfret 2.0 was incubated with increasing amounts of nicotinamide adenine nucleotides, normalized FRET ratios were calculated from emission spectra. NAD: red; NADH: green; NADPH: black. Data and error bars correspond to mean ± SEM (n=4). **(d)** The curves in (c) are re-plotted taking into account the AMP contamination in NAD according to the HPLC analysis in (b). NAD: red; NADH: green and NADPH: black. Data and error bars correspond to mean ± SEM (n=4).

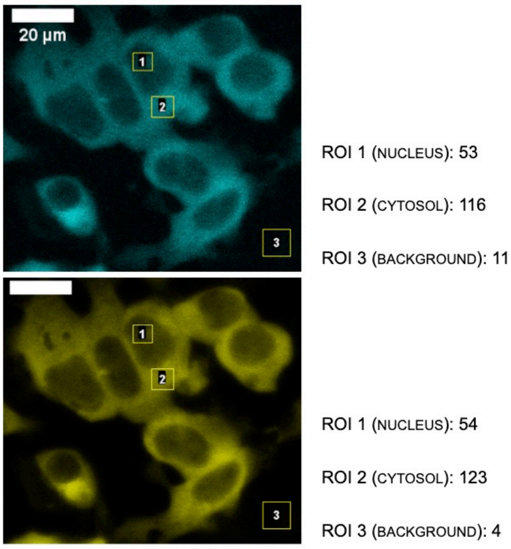

Supplementary Figure 7 Fluorescence in CFP (mseCFPΔ11, top) and YFP channels (cp173Venus, bottom) recorded by confocal microscopy in HEK293t cells transfected with AMPfret 2.1 and maintained under baseline conditions (scale bars: 20 µm). Relative values for exemplary regions of interest (ROI) are given.

Supplementary Table1: Overview of AMPfret constructs tested for 991 responses

AMPfret construct	Organization	FRET change†
AMPfret A (AMPfret pre-2.0)	α_2-CFP _ β_2-YFP _ γ_1	1.010 ± 0.010
AMPfret deletion	$\alpha_{2\text{-AR}}$-CFP _ $\beta_{2\text{-KPI}}$-YFP _ γ_1	1.041 ± 0.013
AMPfret A 211	α_2-CFP _ β_1-YFP _ γ_1	1.022 ± 0.011
AMPfret B	CFP-α_2 _ β_2-YFP _ γ_1	1.026 ± 0.005
AMPfret C (AMPfret 1.0)	α_2-CFP _ β_2 _ γ_1-YFP	*n.d.*
AMPfret C deletion	$\alpha_{2\text{-AR}}$-CFP _ β_2 _ $\gamma_{1\text{-LTGGEKKP}}$-YFP	0.998 ± 0.024
AMPfret C helix	α_2-helix-CFP _ β_2 _ $\gamma_{1\text{-LTGGEKKP}}$-YFP	1.008 ± 0.014
AMPfret C 211	α_2-CFP _ β_1 _ γ_1-YFP	1.003 ± 0.013
AMPfret D	α_2-CFP _ β_2 _ YFP-γ_1	*n.d.*
AMPfret E	CFP-α_2 _ β_2 _ γ_1-YFP	1.006 ± 0.011
AMPfret F (AMPfret CTL)	CFP-α_2 _ β_2 _ YFP-γ_1	0.998 ± 0.009
AMPfret G	α_2 _ β_2-YFP _ γ_1-CFP	*n.d.*
AMPfret H	α_2 _ YFP-β_2 _ γ_1-CFP	*n.d.*
AMPfret I	α_2 _ β_2-YFP _ CFP-γ_1	0.988 ± 0.005
AMPfret I.1	α_2 _ β_2-cpVenus _ mseCFP$_{\Delta 11}$-γ_1	1.001 ± 0.020
AMPfret J	α_2 _ YFP-β_2 _ CFP-γ_1	0.984 ± 0.016
AMPfret J.1	α_2 _ cpVenus-β_2 _ mseCFP$_{\Delta 11}$-γ_1	0.994 ± 0.016
AMPfret K	α_2-CFP _ YFP-β_2 _ γ_1	1.001 ± 0.010
AMPfret K.1	α_2-mseCFP$_{\Delta 11}$ _ cpVenus-β_2 _ γ_1	1.024 ± 0.012
AMPfret L	CFP-α_2 _ YFP-β_2 _ γ_1	1.080 ± 0.019
AMPfret L.1	mseCFP$_{\Delta 11}$-α_2 _ cpVenus-β_2 _ γ_1	1.003 ± 0.009
AMPfret 2.0	α_2-helix-CFP _ β_2-YFP _ γ_1	1.078 ± 0.009
AMPfret 1.1	α_2-mseCFP$_{\Delta 11}$ _ β_2 _ γ_1-cpVenus	1.039 ± 0.014
AMPfret 2.1	α_2-helix-mseCFP$_{\Delta 11}$ _ β_2-cpVenus _ γ_1	1.164 ± 0.004

† Change in FRET ratio (YFP/CFP) was measured in presence of 2 µM compound 991 and normalized to the FRET ratio measured in absence of the compound. Normalized FRET ratios are given as mean ± SEM of at least 3 independent measurements. CTL, control.

Supplementary Table 2: List of primers

Cloning of AMPfret 1st generation

- ECFP for alpha tagging at C-Ter

5' HindIII eCFP:	TTATAAAGCTTATGGTGAGCAAGGGCGAGGAG
3' eCFP Xho1:	AATATCTCGAGTTATCAGTGAGCTCGTCGAGATCTGAGTCC

- ECFP for alpha tagging at N-Ter

5'His10TEVNde1eCFP:	GAAAACCTGTATTTTCAGGGGCATATGGTGAGCAAGGGCGAGGAG
3'eCFP 2Gly a2:	CGTGCTTCTGCTTCTCAGCCATACCGCCGTGAGCTCGTCGAGATCTGAGTCC

- YFP for beta tagging at C-Ter

5'Apal1 YFP:	TTATAGTGCACATGGTGAGCAAGGGCGAGGAG
3' YFP Sph1:	AATATGCATGCTTATCATCTAGATCCGGTGGATCCCGGG

- YFP for beta tagging at N-Ter

5'Nde1-YFP:	TTATACATATGGTGAGCAAGGGCGAGGAG
3' YFP- Stu1:	AATATAGGCCTTCTAGATCCGGTGGATCCCGGG

- CFP for gamma tagging at C-Ter

5'Nhe1 eCFP:	AAAAAGCTAGCATGGTGAGCAAGGGCGAGGAG
3'eCFP Spe1 pDS:	GGCACATGGGTTTAAACGGAACTAGTTATCAGTGAGCTCGTCGAGATCTGAGTCC

- CFP for gamma tagging at N-Ter

5' pDS Nde1-eCFP:	AACTTTAAGAAGGAGATATACATATGGTGAGCAAGGGCGAGGAG
3' eCFP Nhe1:	AAAAAGCTAGCGTGAGCTCGTCGAGATCTGAGTCC

- YFP for gamma tagging at C-Ter

5' Nhe1 YFP:	AAAAAGCTAGCATGGTGAGCAAGGGCGAGGAG
3' YFP Spe1 pDS:	GGCACATGGGTTTAAACGGAACTAGTTATCATCTAGATCCGGTGGATCCCGGG

- YFP for gamma tagging at N-Ter

5' pDS Nde1 YFP:	AACTTTAAGAAGGAGATATACATATGGTGAGCAAGGGCGAGGAG
3' YFP Nhe1:	AAAAAGCTAGCTCTAGATCCGGTGGATCCCGGG

Cloning of mutant (AMPfretC derived)

5' T172A:	GCTAGCTGTGGATCGCCAAATTATGC
3' T172A:	TTTGGCGATCCACAGCTAGCTCGTAGAAATTCACCATCTGACATCATATTAGAG
5' T172D:	GACAGCTGTGGATCGCCAAATTATGC
3' T172D:	TTTGGCGATCCACAGCTGTCTCGTAGAAATTCACCATCTGACATCATATTAGAG
5' L128D + V129D:	GATGATTGCATTTCTCCAAATGCCAGCTTGTTCGATG
3' L128D + V129D:	TTTGGAGAAATGCAATCATCTGGCTTAAAGGAGTCCTGCAGGTAGACCTC
5' V275G + L276G:	GGAGGTAAGTGCTACCTACATGAGACTCTCGAGGCAATCATCAATAGACTG
3' V275G + L276G:	TGTAGGTAGCACTTACCTCCACCCTCGAAGTAGTGTGACCGGTGC
5' S315P:	CCCGATATCTTACAGGCTCTGGTGCTCACAGGTGG
3' S315P:	AGAGCCTGTAAGATATCGGGCAGCGATACAATGCCCTTGACCACGTCATG

Helix

FOR-6AA:	TAGAGGAGGAAGAGAAGAAAGGCGAGGAGCTGTTCACCGGG
REV-6AA:	TTTCTTCTCTTCCTCCTCTAAAGCAGTGATAAGACTGGC
FOR-4AA:	CTGCTTTAGAGGAGGAAGAGGGCGAGGAGCTGTTCACCGGG
REV-4AA:	CTCTTCCTCCTCTAAAGCAGTGATAAGACTGGCG
FOR-2AA:	TTATCACTGCTTTAGAGGAGGGCGAGGAGCTGTTCACCGGG
REV-2AA:	CTCCTCTAAAGCAGTGATAAGACTGGCGCACATTTC
FOR-10AA:	AGAAGAAAAAGAAAGAGGAAGGCGAGGAGCTGTTCACCGGG
REV-10AA:	TTCCTCTTTCTTTTTCTTCTCTTCCTCCTCTAAAGCAGTGATAAGACTGG
FOR-12AA:	AAAAGAAAGAGGAAGAGGAAGGCGAGGAGCTGTTCACCGGG
REV-12AA:	TTCCTCTTCCTCTTTCTTTTTCTTCTCTTCCTCCTCTAAAGCAGTGATAAGACTGG
FOR-14AA:	AAGAGGAAGAGGAAAAGAAGGGCGAGGAGCTGTTCACCGGG
REV-14AA:	CTTCTTTTCCTCTTCCTCTTTCTTTTTCTTCTCTTCCTCCTCTAAAGCAGTGATAAGACTGG
FOR-8AA:	GAGGAGGAAGAGAAGAAAAAGAAA GGCGAGGAGCTGTTCACCGG
REV-8AA:	TTTTTCTTCTCTTCCTCCTC TAAAGCAGTGATAAGACTGGCGCACATTTC

Unfolded Amino Acids deletion

5’ a2-AR: CCAGTCTTATCACTGCTTTA ATGGTGAGCAAGGGCGAGGAG
3’ a2-AR: TAAAGCAGTGATAAGACTGGCGCACATTTC
5’ b2-KPI: ATGTTACTACTCTGCTATAC ATGGTGAGCAAGGGCGAGGAG
3’ b2-KPI: GTATAGCAGAGTAGTAACATACTTCTTCTTGTAGCGATGG

5’ g1-LTGGEKKP: ACATCTTACAGGCTCTGGTG ATGGTGAGCAAGGGCGAGGAG
3’ g1-LTGGEKKP: CACCAGAGCCTGTAAGATGTCAGACAGC

FP exchange

5’Apal1 cpVenus: TTATAGTGCACATGGGCGGCGTGCAGCTCG
3’cpVenus Sph1: AATATGCATGCTCATTACTCGATGTTGTGGCGGATCTTGAAGTTGG
5’ Nhe1 cpVenus: ATTATAGCTAGCATGGGCGGCGTGCAGCTCG
3’Pme1 cpVenus: TTATAGTTTAAACCTATTACTCGATGTTGTGGCGGATCTTGAAGTTGG
5’HindIII CFP trunc: ATATTAAGCTT ATGGTGAGCAAGGGCGAGGAGCTG
3’ Xho1 CFP trunc: TTATA CTCGAGCTATTA GGCGGCGGTCACGAACTCCAG

Cloning in MultiMAM expression system

5’Mam2a2: AGACTTGATCACCCGGGATC ATGGCTGAGAAGCAGAAGCACGAC
3’ a2 helix CFP: TTTTTCTTCTCTTCCTCCTC TAAAGCAGTGATAAGACTGGCGCACATTTC
5 a2 helix CFP: GAGGAGGAAGAGAAGAAAAAGAAA GGCGAGGAGCTGTTCACCGG
3’mseCFP Mam2: CTGCTAGCACCATGGCTCGA CTATTAGGCGGCGGTCACGAACTC
5’MDK b2: AGACTTGATCACCCGGGATC ATGGGAAACACCACCAGCGAC
3’b2 –KPI: GTATAGCAGAGTAGTAACATACTTCTTCTTGTAGC
5’b2 VENUS: ATGTTACTACTCTGCTATAC ATGGGCGGCGTGCAGC
3’ VENUS MDK: CTGCTAGCACCATGGCTCGATCATTACTCGATGTTGTGGCGGATC
5’MDS g1: AGACTTGATCACCCGGGATC ATGGAGTCGGTTGCTGCAGAGAG
3’g1 MDS: CTGCTAGCACCATGGCTCGA TCAGGGCTTCTTCTCTCCACCTG
