# Supplementary material for: Synthetic energy sensor AMPfret deciphers adenylate-dependent AMPK activation mechanism
Source: Nat Commun. 2019 Mar 4;10:1038. doi: 10.1038/s41467-019-08938-z (PMC6399333; doi:10.1038/s41467-019-08938-z)
Supplement: Supplementary file 3 — Reporting Summary [file 41467_2019_8938_MOESM3_ESM.pdf]

nature research

Corresponding author(s): Uwe SCHLATTNER & Imre BERGER

Last updated by author(s): Jan 30, 2019

Reporting Summary

Nature Research wishes to improve the reproducibility of the work that we publish. This form provides structure for consistency and transparency in reporting. For further information on Nature Research policies, see Authors & Referees and the Editorial Policy Checklist.

Statistics

For all statistical analyses, confirm that the following items are present in the figure legend, table legend, main text, or Methods section.

n/a	Confirmed	
☐	☒	The exact sample size (*n*) for each experimental group/condition, given as a discrete number and unit of measurement
☐	☒	A statement on whether measurements were taken from distinct samples or whether the same sample was measured repeatedly
☐	☒	The statistical test(s) used AND whether they are one- or two-sided<br>*Only common tests should be described solely by name; describe more complex techniques in the Methods section.*
☒	☐	A description of all covariates tested
☐	☒	A description of any assumptions or corrections, such as tests of normality and adjustment for multiple comparisons
☐	☒	A full description of the statistical parameters including central tendency (e.g. means) or other basic estimates (e.g. regression coefficient) AND variation (e.g. standard deviation) or associated estimates of uncertainty (e.g. confidence intervals)
☒	☐	For null hypothesis testing, the test statistic (e.g. *F*, *t*, *r*) with confidence intervals, effect sizes, degrees of freedom and *P* value noted<br>*Give P values as exact values whenever suitable.*
☒	☐	For Bayesian analysis, information on the choice of priors and Markov chain Monte Carlo settings
☒	☐	For hierarchical and complex designs, identification of the appropriate level for tests and full reporting of outcomes
☒	☐	Estimates of effect sizes (e.g. Cohen's *d*, Pearson's *r*), indicating how they were calculated

Our web collection on statistics for biologists contains articles on many of the points above.

Software and code

Policy information about availability of computer code

Data collection	FRET data were collected using a fluorometer together with the FeliX32 software (both PTI, Horiba Scientific).<br>Images of living cells expressing the AMPfret biosensor were acquired on Leica SP2 or SP8 confocal microscopes (Leica Microsystems, Germany) using the Leica Confocal Software (Version3.1.1.15751).<br>Immunoblotting membranes and screens exposed to radiolabeled gels were acquired with ImageQuant LAS4000 imager and Typhoon FLA 9000 imager (both GE Healthcare)
Data analysis	Data related to DNA cloning and sequencing were analyzed using Ape-A plasmid editor v2.0.51.<br>FRET data from the PTI fluorometer were analyzed using SigmaPlot 13.0 (Systat Software Inc.) and Excel (Microsoft Office 16).<br>Data from SP2 or SP8 confocal microscopes were extracted using ImageJ (Version1.51g) and analyzed with SigmaPlot 13.0 (Systat Software Inc.) and/or Excel (Microsoft Office16).<br>Images acquired with ImageQuant and Typhoon imagers were analyzed using ImageJ (Version1.51g).

For manuscripts utilizing custom algorithms or software that are central to the research but not yet described in published literature, software must be made available to editors/reviewers. We strongly encourage code deposition in a community repository (e.g. GitHub). See the Nature Research guidelines for submitting code & software for further information.

Data

Policy information about availability of data

All manuscripts must include a data availability statement. This statement should provide the following information, where applicable:

- Accession codes, unique identifiers, or web links for publicly available datasets
- A list of figures that have associated raw data
- A description of any restrictions on data availability

Data supporting the findings of this manuscript are available from the corresponding authors upon reasonable request. A reporting summary for this Article is available as a Supplementary Information file.

Field-specific reporting

Please select the one below that is the best fit for your research. If you are not sure, read the appropriate sections before making your selection.

☒ Life sciences ☐ Behavioural & social sciences ☐ Ecological, evolutionary & environmental sciences

For a reference copy of the document with all sections, see nature.com/documents/nr-reporting-summary-flat.pdf

Life sciences study design

All studies must disclose on these points even when the disclosure is negative.

Sample size	No sample size calculation was performed in this study. In vitro experiments were independently performed at least 3 times. In experiments with living cells, at least 10 cells were measured within at least two independent experiments.
Data exclusions	Data points distant by more than 3 standard deviations from the mean were considered as aberrant and excluded from analysis.
Replication	All FRET experiments are performed in at least three technical replicates and key experiments performed in biological duplicates. Biological replicates of experiments analyzed with WB were performed at least twice.
Randomization	not applicable
Blinding	not applicable

Reporting for specific materials, systems and methods

We require information from authors about some types of materials, experimental systems and methods used in many studies. Here, indicate whether each material, system or method listed is relevant to your study. If you are not sure if a list item applies to your research, read the appropriate section before selecting a response.

Materials & experimental systems

n/a	Involved in the study	
☐	☒	Antibodies
☐	☒	Eukaryotic cell lines
☒	☐	Palaeontology
☒	☐	Animals and other organisms
☒	☐	Human research participants
☒	☐	Clinical data

Methods

n/a	Involved in the study	
☒	☐	ChIP-seq
☒	☐	Flow cytometry
☒	☐	MRI-based neuroimaging

Antibodies

Antibodies used	AMPKα Antibody #2532 (Cell Signaling Technologies Inc)<br>Phospho-AMPKα (Thr172) (40H9) Rabbit mAb #2535 (Cell Signaling Techologies Inc)<br>Acetyl-CoA Carboxylase Antibody #3662 (Cell Signaling Technologies Inc)<br>Phospho-Acetyl-CoA Carboxylase (Ser79) Antibody #3661 (Cell Signaling Technologies Inc)
Validation	AMPKα antibody has been used and validated in doi: 10.1371/journal.pone.0197973<br>Phospho-AMPKα (Thr172) has been used and validated in doi: 10.3892/ijmm.2018.3599<br>ACC antibody has been used and validated in doi: 10.1074/jbc.RA117.001327<br>Phospho ACC antibody has been used and validated in doi: 10.1074/jbc.RA117.001327

Eukaryotic cell lines

Policy information about cell lines

Cell line source(s)	HeLa cells were a kind gift from Prof. Peter Cullen (University of Bristol, UK)<br>3T3L1 cells were purchased at ATCC, (3T3-L1 ATCC® CL-173)<br>HEK293t cells were purchased at ATCC (293T, ATCC® CRL-3216)
Authentication	HeLa were used in DOI: 10.1038/s41467-018-06114-3 and DOI: 10.1038/ncb3610 and characterized in Gey GO, et al. Tissue culture studies of the proliferative capacity of cervical carcinoma and normal epithelium. Cancer Res. 12: 264-265, 1952.<br>3T3L1 were characterized in PubMed ID: 4426090 (Green H, Meuth M. An established pre-adipose cell line and its differentiation in culture. Cell 3: 127-133, 1974.)<br>HEK293T were described and characterized in Pubmed ID: 3031469 (DuBridge RB, et al. Analysis of mutation in human cells

by using an Epstein-Barr virus shuttle system. Mol. Cell Biol. 7: 379-387, 1987) and 7690960 (Pear WS, et al. Production of high-titer helper-free retroviruses by transient transfection. Proc. Natl. Acad. Sci. USA. 90: 8392-8396, 1993.)

Mycoplasma contamination	Hela, 3T3-L1 and HEK293T cells lines were negative for mycoplasma
Commonly misidentified lines (See ICLAC register)	not applicable
